# Supplementary material for: Large mechanical properties enhancement in ceramics through vacancy-mediated unit cell disturbance
Source: Nat Commun. 2023 Dec 16;14:8387. doi: 10.1038/s41467-023-44060-x (PMC10725508; doi:10.1038/s41467-023-44060-x)
Supplement: Supplementary file 1 — Supplementary Information [file 41467_2023_44060_MOESM1_ESM.pdf]

Supplementary Information

Large mechanical properties enhancement in ceramics through vacancy-mediated unit cell disturbance

Zhuo Chen[1], Yong Huang[1], Nikola Koutná[2,3], Zecui Gao[2], Davide G.Sangiovanni[3], Simon Fellner[1], Georg Haberfehlner[4], Shengli Jin[5], Paul H.Mayrhofer[2], Gerald Kothleitner[4,6], Zaoli Zhang[1,7]*

[1]Erich Schmid Institute of Materials Science, Austrian Academy of Sciences, A-8700 Leoben, Austria

[2]Institute of Materials Science and Technology, TU Wien, A-1060 Vienna, Austria

[3]Department of Physics, Chemistry, and Biology (IFM), Linköping University, Linköping SE-58183, Sweden

[4] Institute of Electron Microscopy and Nanoanalysis, Graz University of Technology, Steyrergasse 17, 8010, Graz, Austria

[5] Chair of Ceramics, Montanuniversität Leoben, Peter-Tunner Strasse 5, 8700 Leoben, Austria

[6] Graz Centre for Electron Microscopy, Steyrergasse 17, 8010, Graz, Austria

[7] Department of Materials Science, Montanuniversität Leoben, Franz-Josef-Strasse 18, 8700 Leoben, Austria

*Corresponding author. Email:zaoli.zhang@oeaw.ac.at

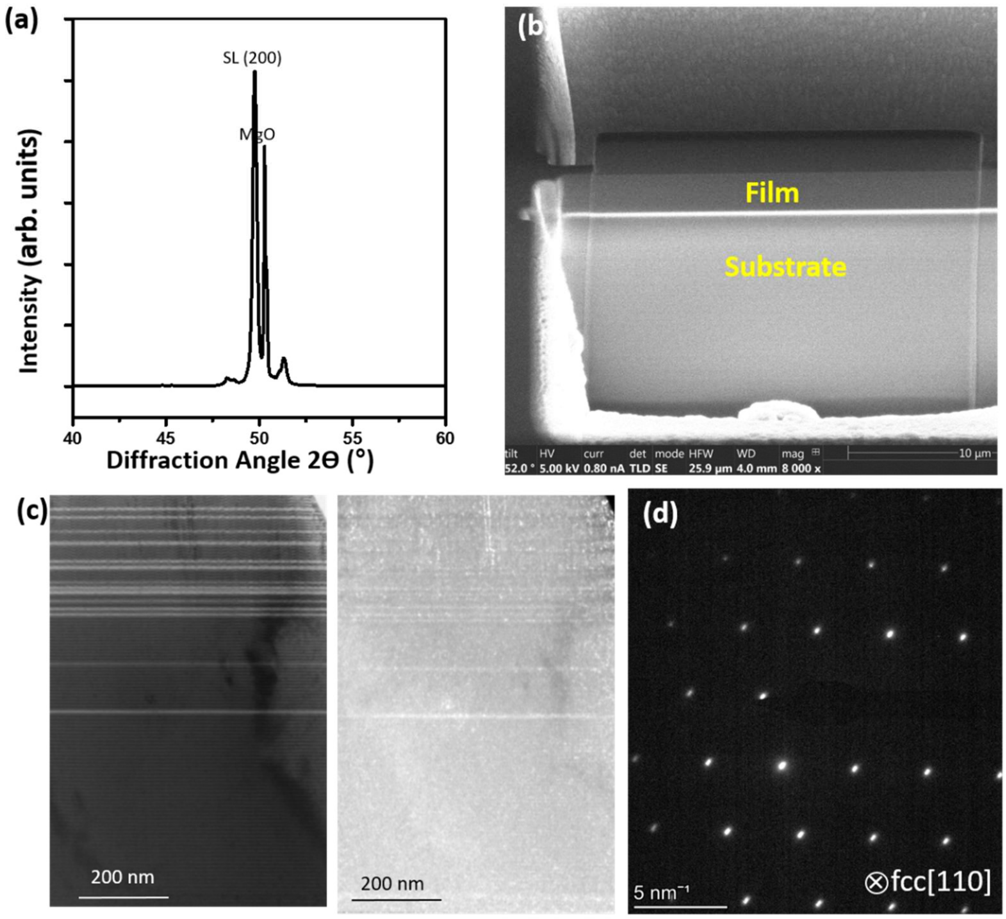

Supplementary Fig. 1: Overall structural observation and phase composition.

(a) X-ray diffraction patterns of SL coating deposited on MgO (100). **(b)** SEM micrographs of SL cross-sections. **(c)** Overall TEM-BF/DF observation of as-deposited SL. **(d)** SAED result of as-deposited SL. The aperture diameter is about 1.0 µm, which contains most of the film (film total thickness is about 1.4 µm). The XRD patterns of TiN/WN_x coatings grown on MgO (100) depict a clear monocrystalline (100) texture. SEM cross-sectional and HAADF observations also clearly show that our SL has no grain boundary structure (2D defect) or pores (3D defect). In addition, our large-scale SAED (aperture diameter is about 1.0 µm) results also demonstrate the single-crystal character of the current TiN/WN_x film.

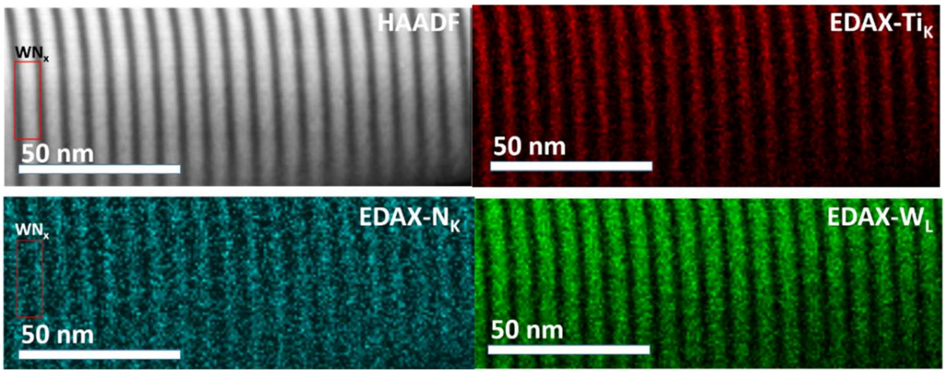

Supplementary Fig. 2: EDXS component analysis of as-deposited SL.

EDXS mappings of $WN_{0.5}$/TiN superlattice. The EDXS mapping demonstrates the N content differences in different layers. The N signal intensities are significantly lower in the $WN_{0.5}$ layer than in

TiN layer (as framed), which to a certain extent, indicates the much higher N vacancy concentration. Note that the curvature in the upper portion of the image is due to the sample drift during data recording.

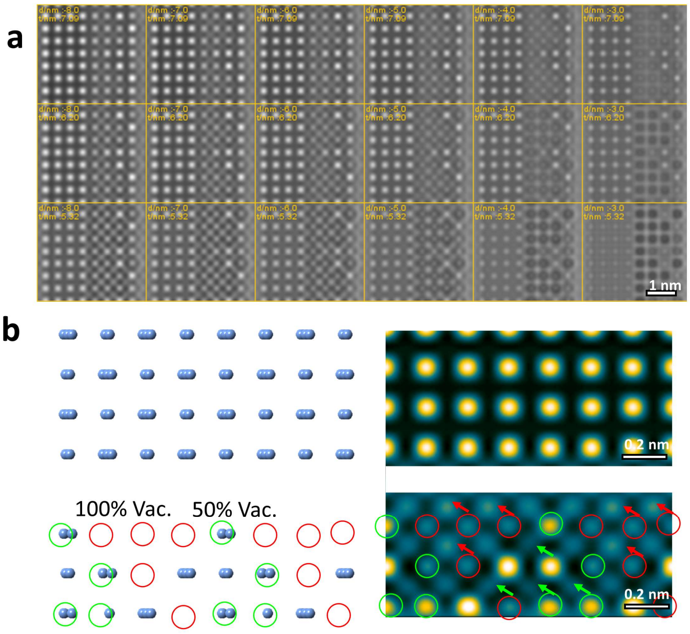

Supplementary Fig. 3: Image simulation of N vacancies in the <100> projection.

(a) Simulated thickness focus map of W_1N_1 and $W_1N_{0.5}$. Note that the simulated images (a) rotate 90° relative to the atomic model (b). The image simulation conditions are: Cs=0 nm, thickness= 5.32-7.09 nm, defocus=-8—-5 nm, defocus spread= 4.8 nm, B2=0 nm, A2=0 nm, Cc=1.2 mm. One part is the W_1N_1 without vacancy (left), and the other part is $W_1N_{0.5}$ (right).

(b) Comparison of atomic model and simulation results. In the atomic model (here only show N position), the columns of atoms marked by green circles have 50% N vacancies, while the columns of atoms marked by red circles have 100% N vacancies. Thus, in the simulation image of the $W_1N_{0.5}$ part, we can see that the W/N atomic column intensity in the <100> projection has irregular variations, i.e., the atomic column with high N vacancy concentration has a lower intensity. Furthermore, we observed extra irregular intensities on (400) planes. This may originate from changes in electron scattering conditions caused by neighboring vacancies. We found that such features are not severe near the low vacancy concentration region (as marked by green arrows in (**b**)), while additional intensities are more strong near the high vacancy concentration region (as marked by red arrows in (**b**)). Therefore, under the current image simulation conditions, the additional intensity is proportionally related to the local vacancy concentration

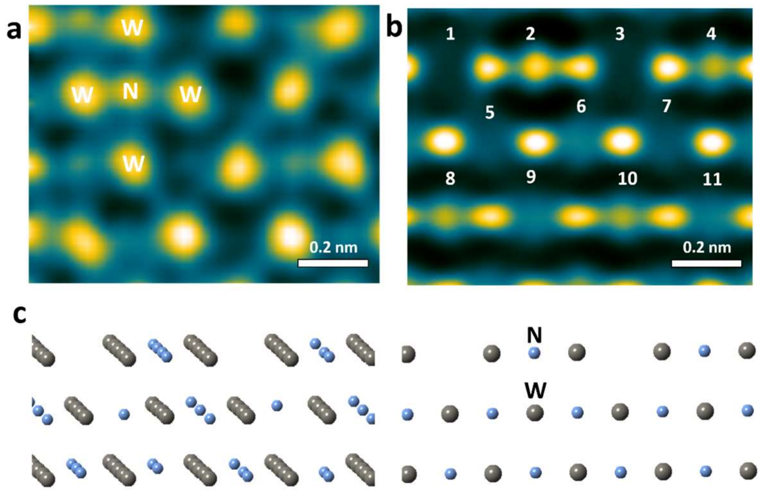

Supplementary Fig. 4: Image simulation of N vacancies in the <110> projection.

(a) and **(b)** Experimental observation and image simulation of N vacancies in the $WN_{0.5}$ layer in the <110> direction. **(c)** Atomic model of $WN_{0.5}$. (oblique projection and exact <110> projection) The image simulation conditions are: Cs = 0nm, thickness= 5nm and defocus = -6 nm defocus spread= 4.8 nm, B2 = 0 nm, A2 = 0 nm, Cc = 1.2 mm. In Fig.S3b, the N atomic columns at different positions have different N concentrations.

According to the simulation model, the N concentrations at positions are set to N1-0%, N2-100%, N3-0%, N4-75%, N5-25%, N6-50%, N7-25%, N8-75%, N9-50%, N10-75%, and N11-50%. The simulated image in (**b**) illustrates that the N column intensities remarkably change with N concentrations (in other words, N vacancy concentrations). The comparison indicates that the N vacancy concentration at the N2 position is the lowest, and the N signal intensity is the highest. Since the imaging conditions of the experimental observation and simulations are quite close, we believe that the N signal intensity (experimental) is directly proportional to the N concentration.

From the atomic model, the N atom concentration is set to be 47.7% (corresponding to a vacancy concentration of 52.3%). Based on the simulated image, we measured the signal intensity of each N atomic column in (**b**), and the average N intensity (of all N atom columns) /maximum N intensity (position N2) is 46.4%, which is almost in agreement with the model N concentration. Therefore, extensive image simulations illustrate that the measurement of the N signal intensity can reflect the actual N concentration of the atomic columns when the thickness is 5.01-7.52 nm.

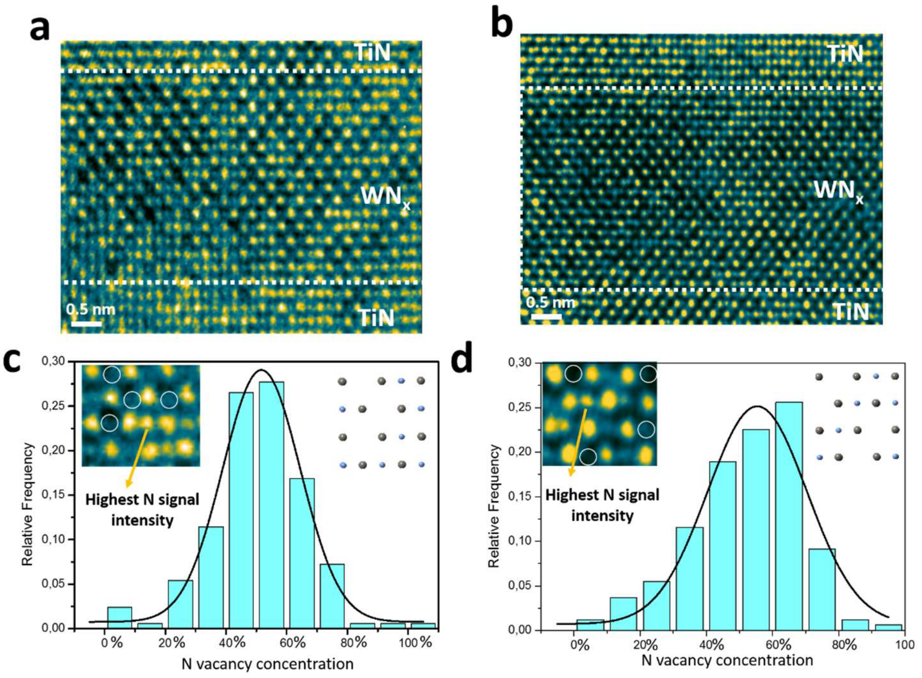

Supplementary Fig. 5: Quantitative statistics of N vacancies.

(a) (b), HRTEM images in WN_x/TiN SL viewing on <110> direction (two images are used for statistical analysis). Due to ideal imaging conditions utilized (Cs~0, sample thickness less than 10 nm, and suitable defocus), W, Ti, and N atomic columns are resolvable in both HRTEM images. It can be seen that the contrast distribution of N atomic columns in WN_x is highly inhomogeneous, and there is almost no N intensity at some column positions. **(c) (d),** Statistical distribution of N vacancy concentration derived from images in (**a**) and (**b**), respectively. Here, we estimate the N concentration by a ratio---the respective intensity/maximum intensity (therefore, vacancy concentration = 1- Intensity/Max-Intensity). Inserts in (**c**) and (**d**) show the maximum N signal intensity observed in the WN_x layer (yellow arrow). Although it is hard to fully confirm that the observed atomic column with the strongest N signal is stoichiometric (i.e., no N Vac.), we find that the maximum N signal intensity in the WNx is close to the maximum N signal intensity in the TiN layer. Therefore, we believe that the observed maximum N signal intensity can be used to evaluate the N vacancy concentration. Here, we evaluate the vacancy concentration from a total of 300 atomic columns and obtain the average N vacancy concentration in (**a**) and (**b**) to be 47.8% (Standard Deviation=15.3%) and 51.9% (Standard Deviation=17.1%), respectively.

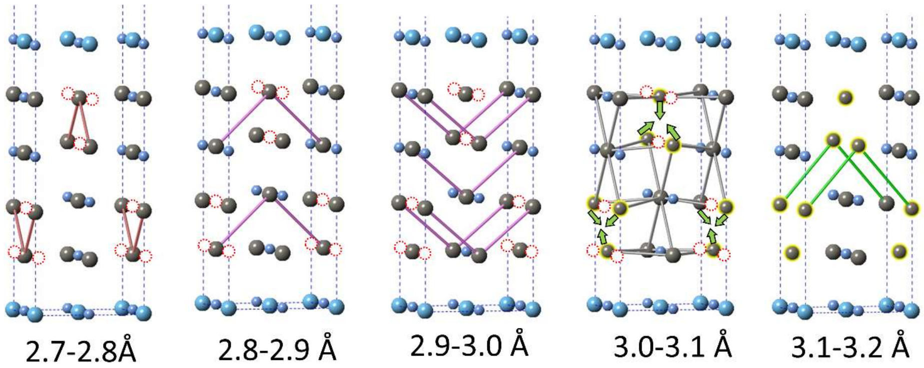

Supplementary Fig. 6: W-W bond length distribution of high-vacancy $WN_{0.5}$.

DFT-simulated $WN_{0.5}$ with disordered N-vacancy structure. Based on the after-relaxation computational model, we found that W-W metallic bonds are being compressed or elongated. The length of the metallic bond is distributed between 2.7 and 3.2Å.

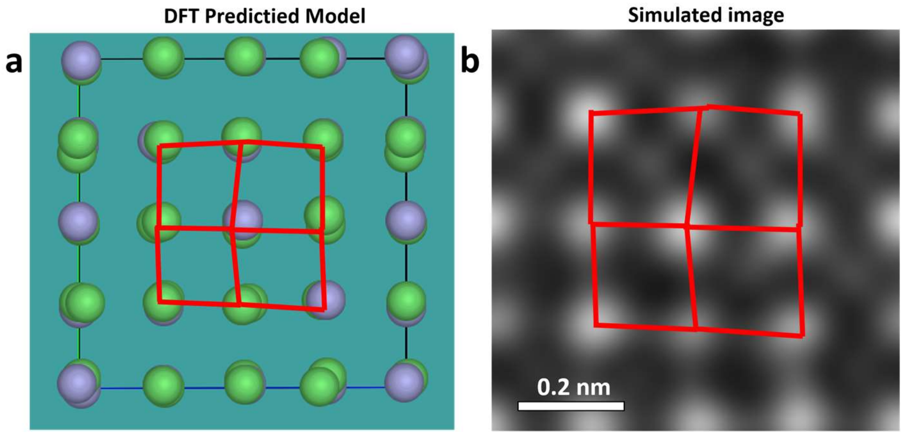

Supplementary Fig. 7: HRTEM images simulate lattice distortion in <100> projection.

HRTEM image simulation (viewing on <100> direction) using DFT predicted model. **(a)** DFT predicted WN_X with 50% disordered N vacancies. **(b)** Simulated HRTEM image. In the simulated image (**b**), additional contrasts appear at some locations (white labels), which is caused by the locally higher concentration of N vacancies. The image simulation conditions are: Cs=0nm, thickness =10 nm and defocus= -9 nm defocus spread= 4.8 nm, B2=0 nm, A2=0 nm, Cc=1.2 mm

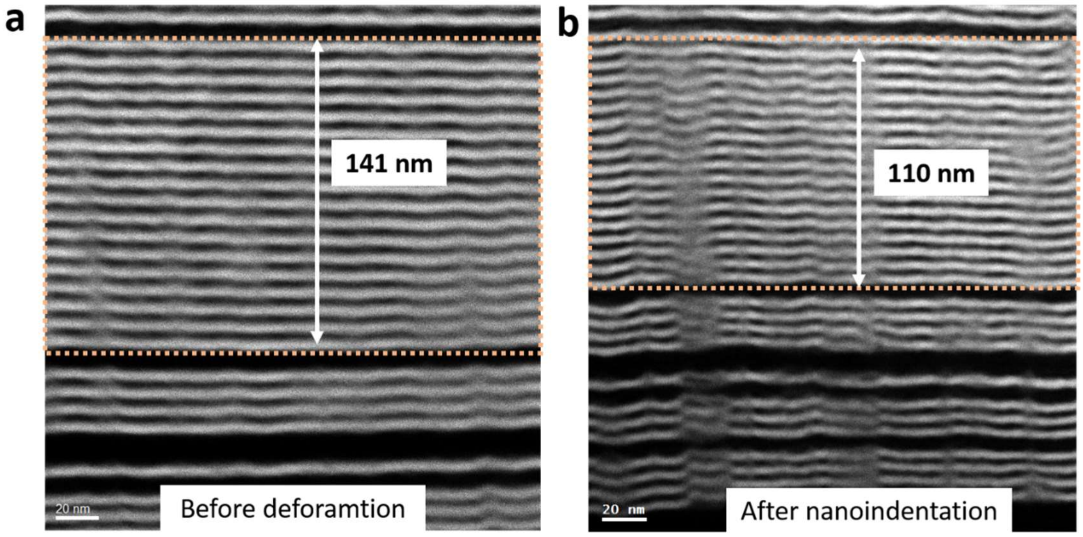

Supplementary Fig. 8: Local compressive strain after deformation.

(a) (b) HAADF observations before and after nanoindentation, respectively. The observed location is about 450-300 nm from the coating surface. After nanoindentation, the thickness of the marked region is reduced to 110 nm from 141 nm before deformation. Therefore, such a local area is received with a compressive strain of 22%. Overall, the indentation tip region (from the coating surface to the substrate interface) has a compressive strain of about 16% (not shown here) under a load of 500 mN.

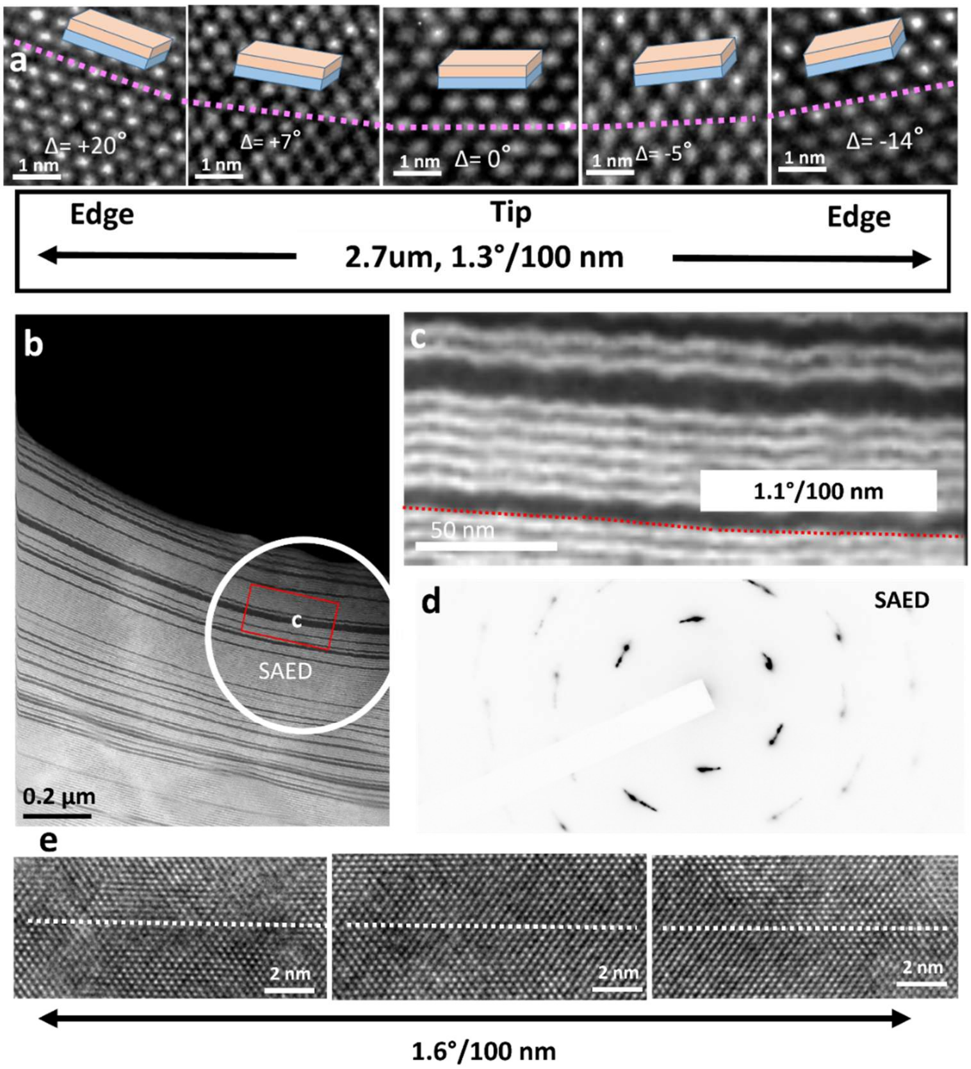

Supplementary Fig. 9: Local lattice rotation after deformation.

(**a**) A series of HRTEM images (recorded from a similar distance away from the impression surface) showed that the bending of the film was accomplished by lattice rotation from +20° to -14° over a range of 2.7 μm, i.e., the average degree of rotation is 1.3°/100 nm. (**b**) A HAADF image shows the overall morphology after nanoindentation. (**c**) HAADF observation at tip region. (**d**) SAED result at tip region. For the near tip region (**c**), we can observe the significant multilayer bending, which indicates a macro-scale bending angle of 5 ° over the 450 nm range (average degree of tilting is 1.1°/100 nm). (**d**) also proves that the macro-scale bending is via a lattice rotation, i.e., forming a 'ring' pattern with diffraction spots spreading. (**e**) A series of HRTEM images present a continuous atomic structure observation of the WN layer over a 90 nm range, which does not show any significant GB structure but only continuous crystal bending. The crystal orientation of each HRTEM image differs by approximately 0.5°. Thus, we show that the lattice rotations are continuous and gradual with a small angle.

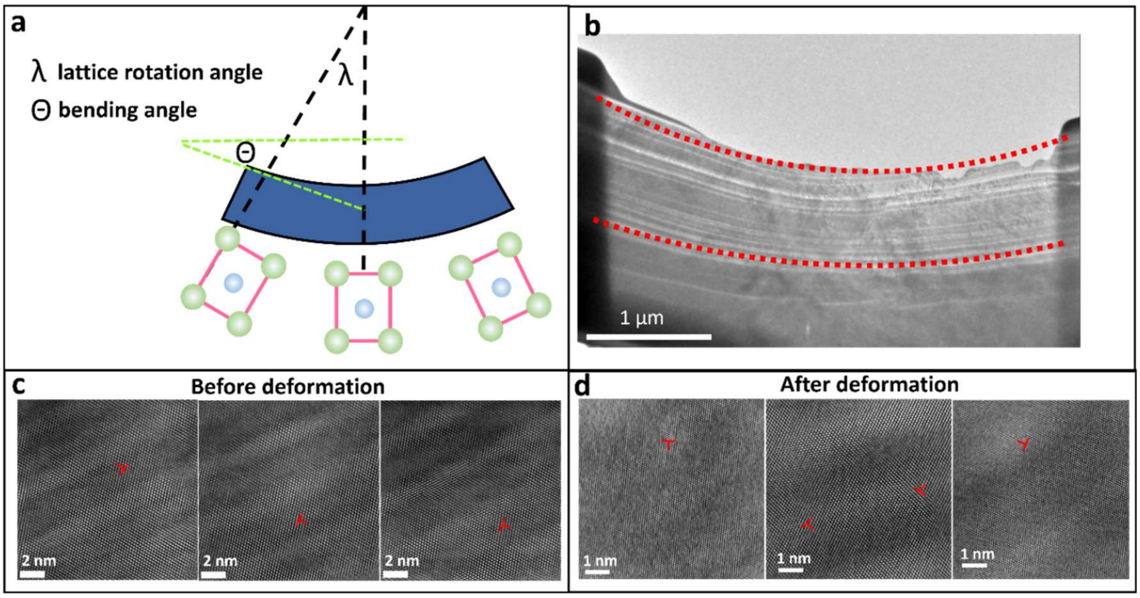

Supplementary Fig. 10: Dislocation density statistics after deformation.

(**a**) Schematic diagram showing the geometric relationship between lattice rotation (λ) and bending angle (θ). (**b**) Overall TEM-BF result of indented SL. **(c)** and **(d)** Observation of dislocation distribution before and after deformation, respectively.

As shown in **(a)**, if plastic deformation is not accomplished via interfacial deformation at all, then the lattice rotation (λ) will be exactly equal to the macroscopic bending angle (θ) [1, 2, 3]. The stored GNDs within the crystal will induce lattice rotation in such a case. Our TEM observation shows that the measured maximum lattice rotation (λ, lattice orientation difference between the edge region and the tip region measured by HRTEM) fully equals the maximum macroscopic bending angle (by overall TEM-BF measurement). According to traditional dislocation theory, this will be achieved with many GNDs.

The locally stored GND density (ρ) can be calculated based on the local curvature η, and the Burgers vector ***b*** uses the following equation[1, 3, 4]:

$$\rho = \frac{\eta}{b} = \frac{1}{bR} \tag{1}$$

Where R is the inverse of local lattice curvature. By fitting the curvature of the bending layers (**b**). We estimate the deformed GND density required by the traditional dislocation theory will increase by 2.03 x $10^{13}/cm^2$ (maximum bending angle with 20°).

In the following, by HRTEM observation, we compare the measured dislocation density before and after deformation. **(c)** and **(d)** show several HRTEM observations before and after deformation (more HRTEM results are not shown here). In **(c)** and **(d)**, we found that the dislocation density before and after deformation did not show a noticeable change. By counting 80 HRTEM images (image size: 17 nm x17 nm), we show that 1/3 {111} type Frank dislocations density is 2.98 x $10^{13}/cm^2$ before deformation and 3.28 x $10^{13}/cm^2$ after deformation. Therefore, our HRTEM statistical analysis shows that the dislocation density after deformation (the net increase) does not reach the theoretical GND density.

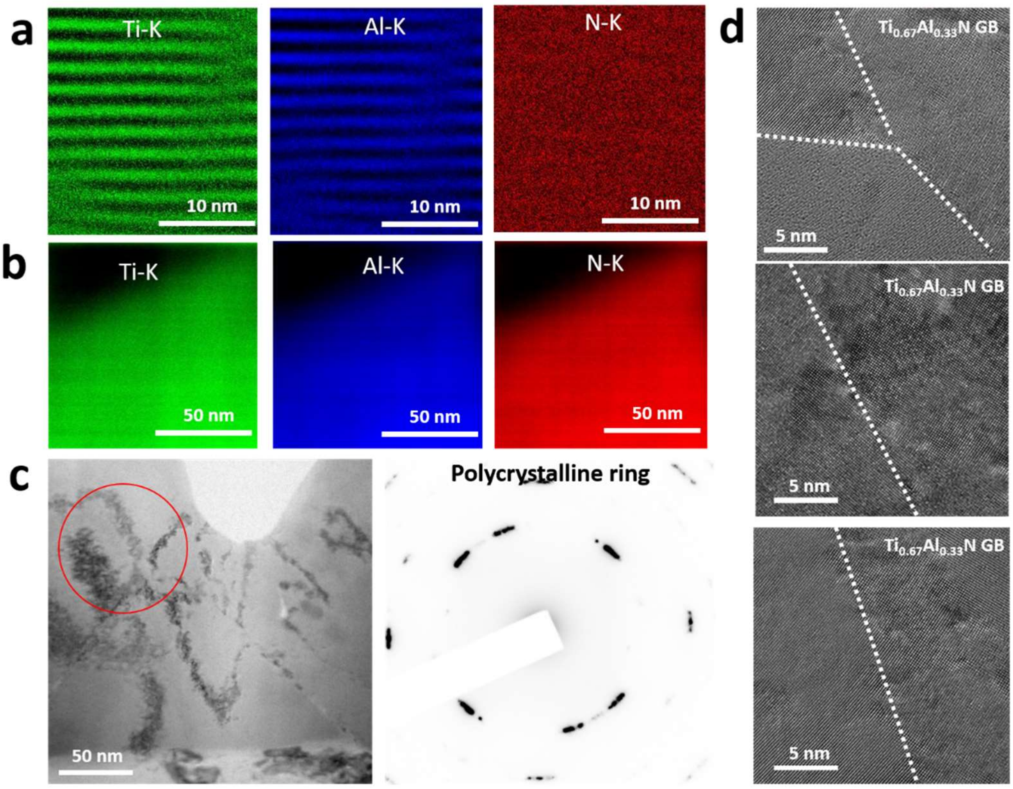

Supplementary Fig. 11: Deformed induced GB structure in TiN/AlN SL.

(a) and **(b)** EDXS mapping of TiN/AlN SL before and after deformation, respectively. **(c)** TEM-BF and SAED results of corresponding areas. **(d)** HRTEM observation of the GB structure. Deformation in a stoichiometric SL. To confirm that the non-stoichiometric $WN_{0.5}$ we observed has unique deformation behaviors, we also performed complementary observations on TiN/AlN SL. Here, TiN/AlN SL with 2.5 nm bilayer thickness. Before deformation, EDXS results (**a**) showed no significant difference in N concentration between TiN and AlN. At the same time, the thermodynamically vacancy-free TiN or AlN is considered a stable structure. Thus, we indicate no significant N vacancies in the TiN/AlN SL, i.e., TiN/AlN is a stoichiometric SL. After deformation, since the SL interface has a large amount of GND, this leads to the mixing of the SL (see details [5, 6]). The surface area of the imprint thus forms a stoichiometric solid solution TiAlN structure (**b**). In these surface solid solution regions, we can find significant plastic deformation. These plastic deformations are mainly the rotation and tilt of the crystal. The SAED result suggests a ring-shaped diffraction feature in the solid solution region (**c**). Hence, within the solid solution region, we can observe the tilt grain boundaries of $Ti_{0.67}Al_{0.33}N_1$ (HRTEM results are shown in (**d**). As seen, such is different from WN_x.

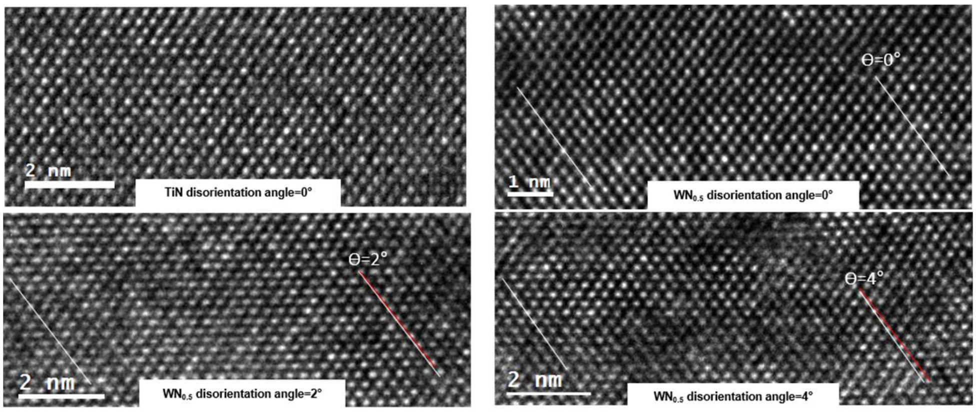

Supplementary Fig. 12: HRTEM observation lattice distortion and crystal bending.

Raw HRTEM images of GPA in Fig.4b. TiN (misorientation angle = 0°) and WN_x layer under different misorientation angles (~0°, ~2° and ~4°). The misorientation angle here is provided by comparing the angle difference between the (111) planes from the left side of the image to the right side of the image. The GPA in Fig.4b was obtained by selecting two non-collinear Bragg vectors ((111)/(11-1)in the power spectrum generated from a high-resolution TEM image.

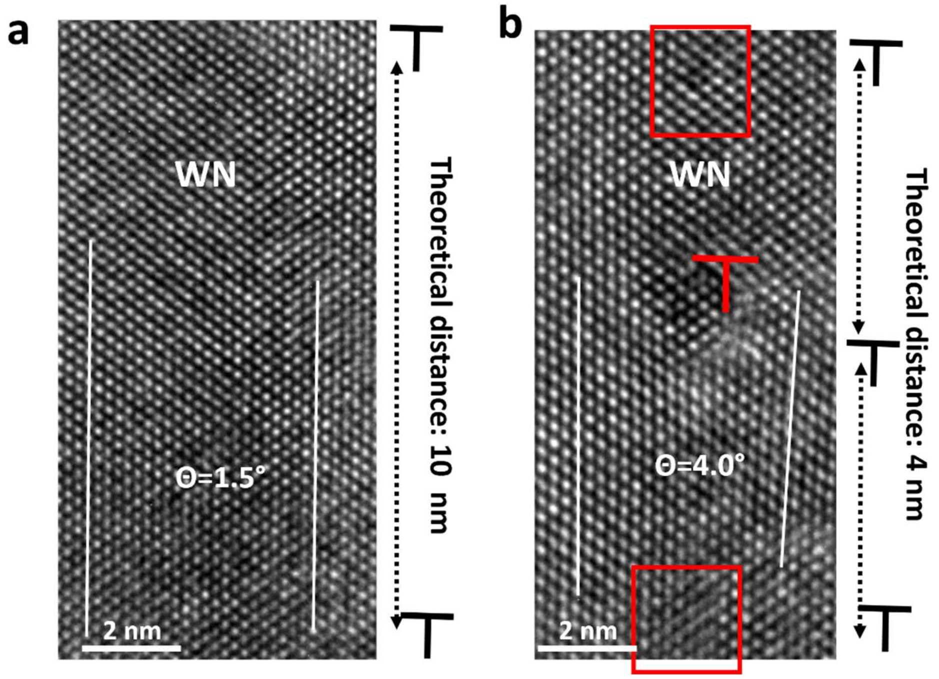

Supplementary Fig. 13: Dislocation spacing measurement at misorientation angle smaller than 4.0°.

(a) and (b), HRTEM observations of $WN_{0.5}$ layers with misorientation angles of 1.5°and 3.5°, respectively. For the 1.5° misorientation angle between two TiN layers (**a**), we did not find any dislocations in the limited observation area (13nm x 6nm). Note that for conventional dislocation theory, when the misorientation angle between crystals is 1.5 °, two dislocations with a 10 nm spacing will be formed. For the 4.0° disorientation angle between two TiN layers (**b**), we only see an isolated dislocation. Theoretically, when the misorientation angle between crystals is 4°, two dislocations with a spacing of 4 nm will be formed. However, in (**b**), the required dislocation is replaced by severe lattice distortion (as labeled by the red box).

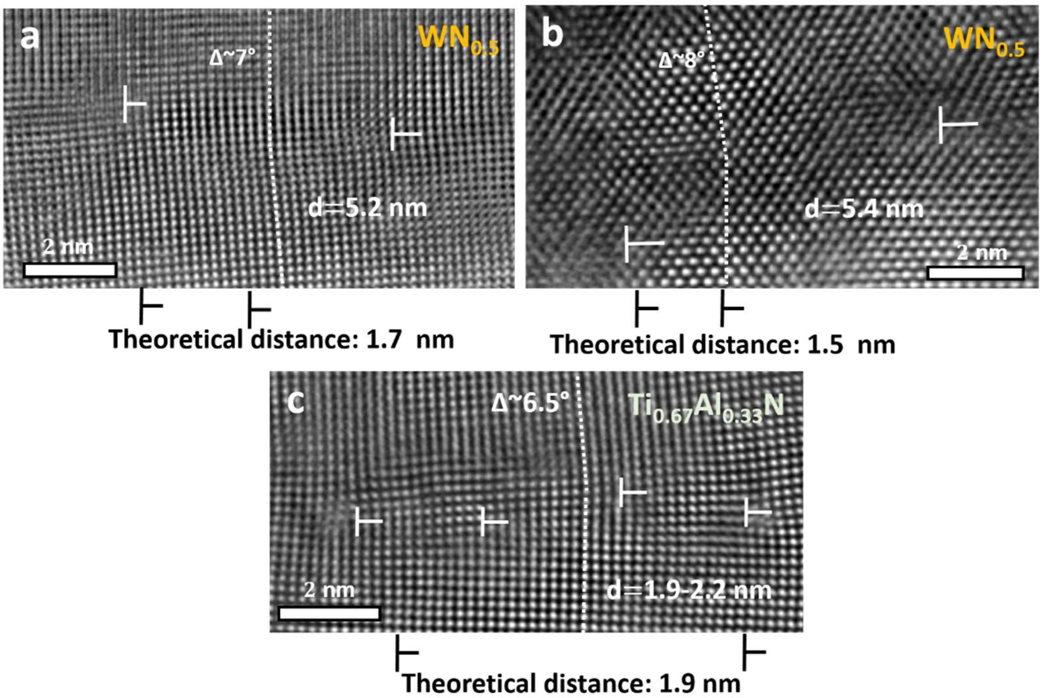

Supplementary Fig. 14: Dislocation spacing measurement at misorientation angle larger than 4.0°.

(a) (b) HRTEM observation of $WN_{0.5}$ layers with misorientation angles of ~7.0° and ~8.0°, respectively. **(c)** HRTEM observation of stoichiometric TMN ($Ti_{0.67}Al_{0.33}N$) layers with misorientation angles of ~6.5°. For the 7.0° misorientation angle between two TiN layers ((**a**), <100> projection), the measured dislocations distance is about 5.2 nm. For the 8.0° disorientation angle between two TiN layers ((**b**), <110> projection directions), the measured dislocation distance is about 5.4 nm. Here, the dislocation distances for both cases are significantly larger than the theoretical dislocation distances derived from the Frank-Bilby equation, which suggests dislocation distances of 1.7 nm and 1.5 nm, respectively. However, for stoichiometric TMN ($Ti_{0.67}Al_{0.33}N$), the relationship between the dislocation distance and the disorientation angle of the experimentally measured follows the theoretical Frank-Bilby equation (as seen in (**c**)).

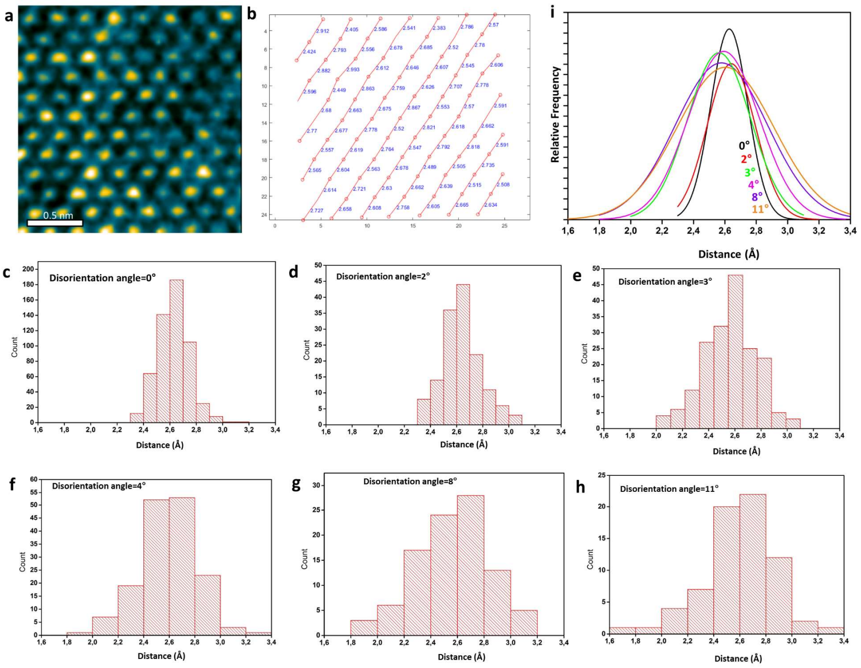

Supplementary Fig. 15: Quantitative statistics of lattice distortion through HRTEM images.

(a) (b) HRTEM image and corresponding atomic column distance distributions. The atomic column spacings were calculated using atomic positions determined by fitting each atom site with a spherical Gaussian function (an algorithm in CalAtom [7]software). (**c**)-(**h**) Statistical distributions of atomic column distances based on different lattice deformation (i.e., 6 HRTEM images with different misorientation angles). Atomic column distances are measured along the <112> direction on the {111} planes. (**i**) The distribution fitting curves (of the histogram (**c**)-(**h**)) at different misorientation angles. Note that the lattice distortion (is described as the standard deviation of atomic spacing, $\overline{u}^{d}$) will increase with the misorientation angle. The standard deviation of atomic spacing $\overline{u}^{d}$ is expressed as:

$$\overline{u}^{d} = \sqrt{\sum_{i=1}^{n}(d-\overline{d})^2/n} \qquad (2)$$

$\overline{d}$ is the average atomic spacings. Since HRTEM is a two-dimensional projection of atomic potential, the standard deviation here may be affected by the TEM sample thickness. Therefore, the HRTEM observation and statistic areas here are chosen from the areas as closed thicknesses.

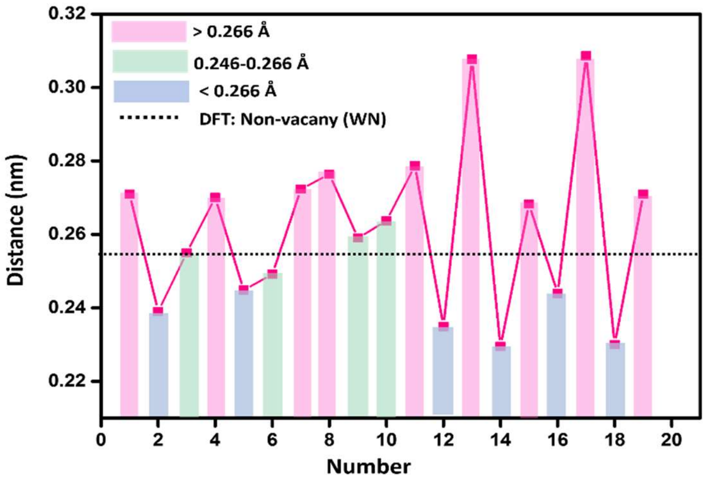

Supplementary Fig. 16: Atomic column distance distribution in $WN_{0.5}$.

Atomic column distances in deformed $WN_{0.5}$, which is measured along the <112> direction on the {111} planes. Line profile results in $WN_{0.5}$ show that atomic column spacings oscillate intensely, indicating an appreciable distortion of the metal sub-lattice.

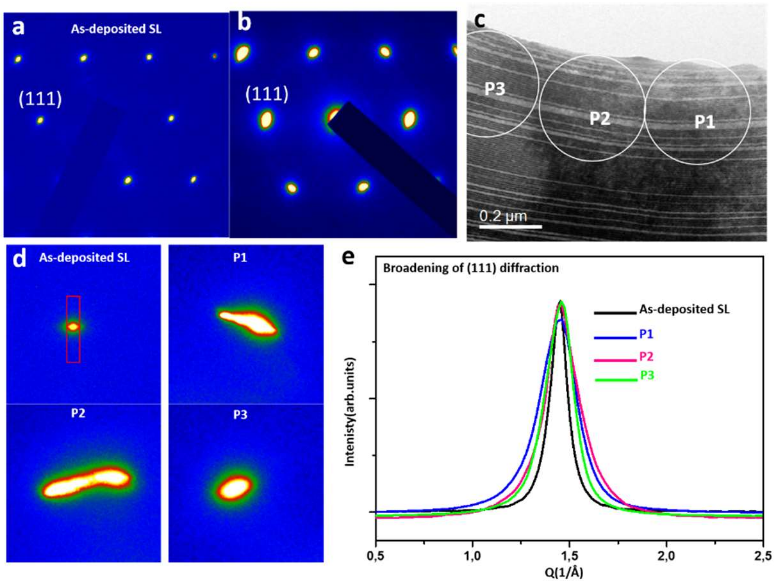

Supplementary Fig. 17: Quantitative analysis of diffraction peak broadening.

(a) (b) SAED result of as-deposited and indented SL, respectively. **(c)** TEM-BF image of indented SL, and three locations (P1, P2, P3) for diffraction analysis. (**d)** Enlarged SAED results from position P1-P3 indicate the broadening of the diffraction spot in (111) reflection. **(e)** The relationship between the reciprocal length and intensity of diffraction spots.

The degree of broadening of the diffraction spot is shown here. The distortion in the crystal lattice (or even in the unit cell) can result in the broadening of diffraction spots. Based on this, we inversely evaluate the distortions by qualitatively analyzing the extent of broadening in the (111) diffraction spot before and after deformation. The broadening of the (111) diffraction spot in the deformed superlattice (SL) is more significant than that observed in as-deposited coating (as shown in **e**). Our HRTEM observations and statistical analysis hardly reveal a significant increase in dislocation density, and interplanar spacing measurements also did not show notable residual strain (not presented here).

Consequently, we, on a larger scale, present evidence of deformation-induced unit cell disturbances. The SAED aperture used was ~500 nm, the camera length was 30 cm, and the acquisition time was 1 s.

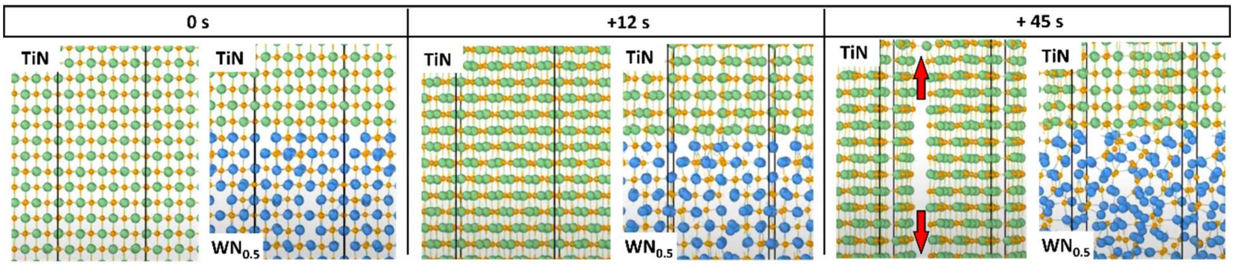

Supplementary Fig. 18: AIMD snapshots during [100] tensile tests.

AIMD snapshots of TiN and WN0.5/TiN SL (≈ 5 nm bilayer thickness) at selected strain steps during [100] tensile tests. Red arrows mark crack opening near dislocations. AIMD simulations indicate that the mechanical response of monolithic TiN is more brittle than WN0.5/TiN SL. With increasing [100]-strain, bonds in the monolithic TiN simply elongate/shorten without changing bond angles between nearest neighbors, and a large void opens at 45 s. Contrarily, void opening in the TiN layer of the SL is retarded (till ~53 s) by irregular distortions in WN0.5, able to flexibly change nearest neighbor bond lengths and angles. AIMD simulations, therefore, point towards lattice distortions as the main carriers of plasticity in $WN_{0.5}$.

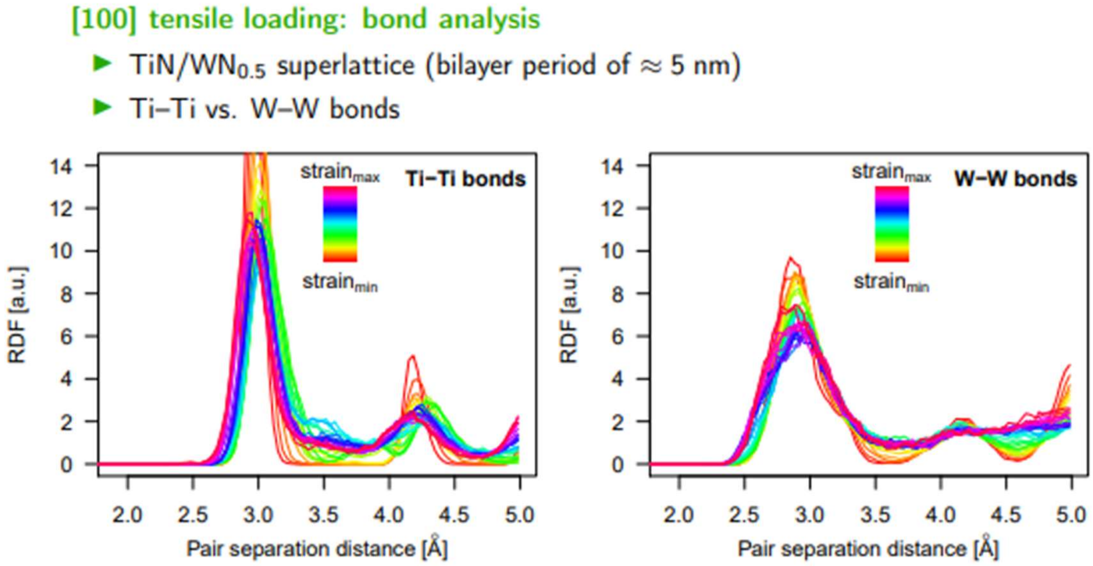

Supplementary Fig. 19: Radial distribution function (RDF) extracted from [100] AIMD tensile loading.

Radial distribution function (RDF) extracted from AIMD simulations of $WN_{0.5}$/TiN SL subject to [100] tensile loading. The color scale marks RDF at different deformation stages, i.e., for increasing [100]-strain. The left and right panels show the RDF of the first and second nearest neighbor Ti-Ti and W-W bonds, respectively. In contrast to sharp and relatively narrow peaks of Ti-Ti bonds at low strains, broader peaks of W-W bonds demonstrate their larger spread. Compared to relatively insignificant changes in Ti-Ti RDF, the W-W RDF peak width becomes more pronounced with increasing strain (especially for the second adjacent bond). Consequently, the degree of lattice distortion in WN0.5 layers notably increases upon [100] tensile straining.

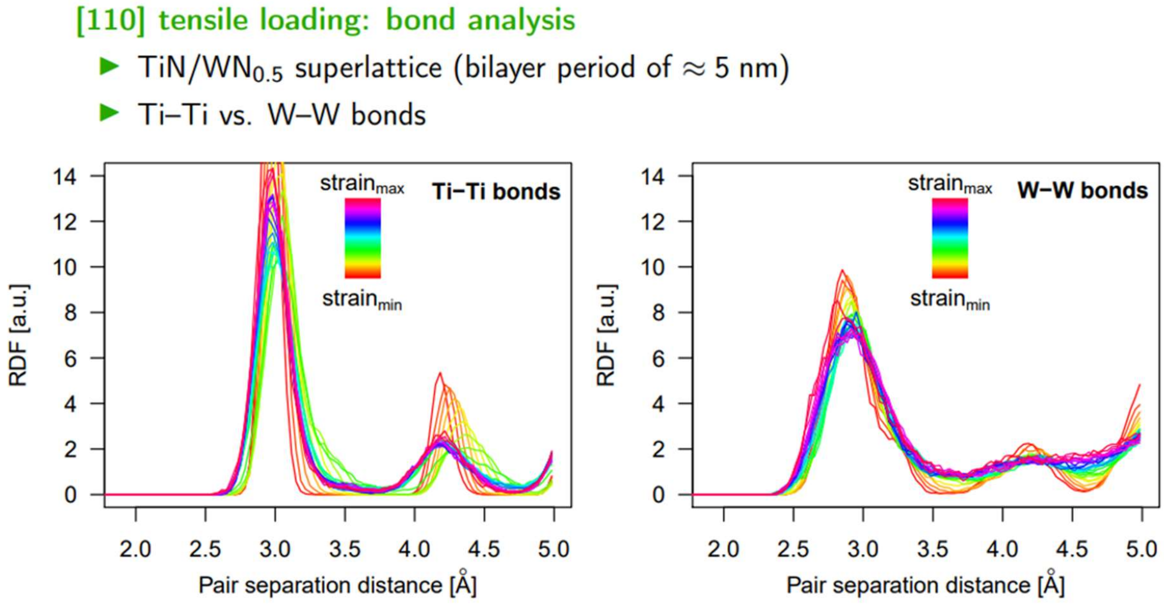

Supplementary Fig. 20: Radial distribution function (RDF) extracted from [110] AIMD tensile loading.

Radial distribution function (RDF) extracted from AIMD simulations of $WN_{0.5}$/TiN SL subject to [110] tensile loading. The color scale marks RDF at different deformation stages, i.e., for increasing [110]-strain. The left and right panels show the RDF of the first and second nearest neighbor Ti-Ti and W-W bonds, respectively. Similar to [110] tensile loading, RDF analysis of [100] deformation shows a wide spread of W-W first and second nearest neighbor bonds under deformation.

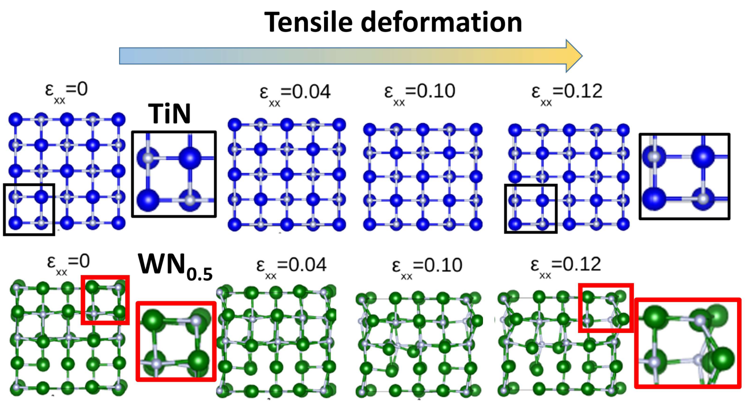

Supplementary Fig. 21: DFT simulation results of monolithic TiN and $WN_{0.5}$ under tensile deformation.

Simulation of stoichiometric TiN subject to [100] tensile elongation at 0K reveals elastic deformation characteristics, with unchanged atomic arrangement and uniformly elongated/shortened nearest neighbor Ti-Ti, Ti-N, and N-N bonds. In $WN_{0.5}$, however, the equivalent elastic strain gives rise to strongly inhomogeneous deformation, where the unit cells exhibit varying degrees of unit cell distortion (as framed), which can act as carriers of elastic deformation in $WN_{0.5}$.

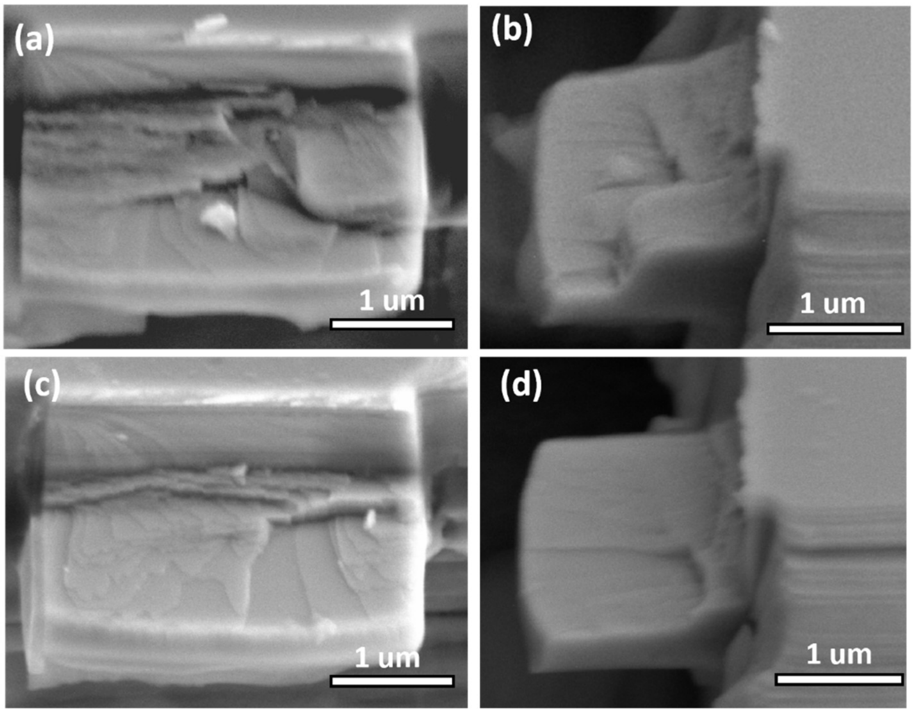

Supplementary Fig. 22: SEM micrographs of fractured cross-sections of cantilever beams (un-notched sample).

(a) and (c) are the top view images, and (b) and (d) are the corresponding side view images, respectively. SEM micrographs of fracture surfaces show stepped features. Since the fracture here is very rough and the crack extends tortuously, this suggests the WN_x SL can significantly deflect the crack.

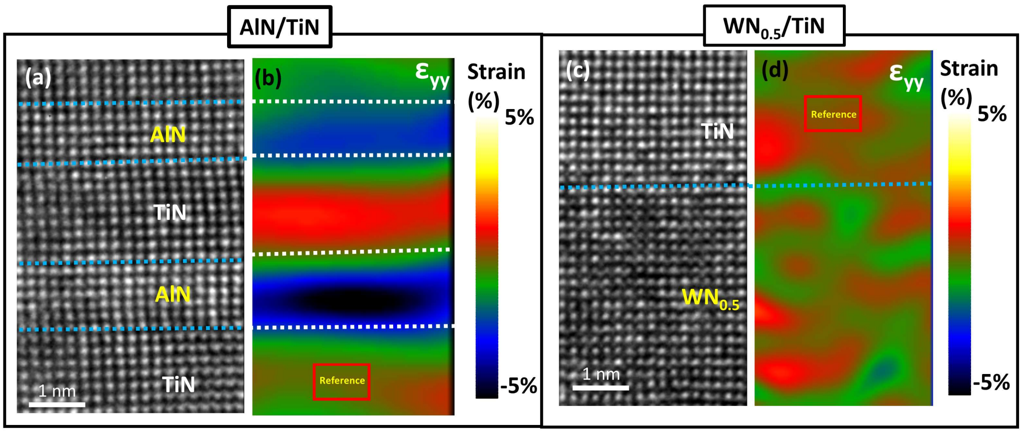

Supplementary Fig. 23: Quantitative analysis and comparison of SL interface strain field.

(a) HRTEM observation (viewed along the [001] direction) of the coherent interface of the as-deposited TiN/AlN SL. **(b)** The corresponding GPA analysis. **(c)** HRTEM observation (viewed along the [001] direction) of the coherent interface of as-deposited TiN/WN_x. **(d)** The corresponding GPA analysis of the image. The red-marked boxes in (b) and (d) are used for reference. The residual stress/strain, i.e., misfit strain, at the coherent interface strongly depends on the different lattice constants of the two layers. For TiN a=4.25Å and $WN_{0.5}$ a=4.18Å[8], the theoretical strain is only about 1.7%. This is much lower than conventional SL, e.g., TiN/AlN is about 3.2%. Our GPA results did not reveal significant strain

differences between the two layers. This indicates that the unit cell disturbances of the $WN_{0.5}$ layer attenuate the elastic stress field.

Supplementary Table1: A summary of the experimentally measured hardness, moduli, and fracture toughness of metal nitride coatings.

All summarized hardness and modulus data are derived from nanoindentation experiments. The fracture toughness of all thin films was unveiled by performing pre-notched single cantilever bending experiments of freestanding coating material. The H/E ratio reveals the empirical indicator of toughness. A non-stoichiometric system ($WN_{0.5}$/TiN) has almost the highest H/E ratio and intrinsic fracture toughness. * are single crystal TMN coatings.

	**Hardness (GPa)**	**Intrinsic fracture toughness (K_{ic}, MPa$\sqrt{m}$)**	**Elastic Modulus (GPa)**	**H/E**
$WN_{0.5}$/TiN* [8]	36.6	4.6	387	0.092
TiN* (MgO-Sub) [8]	31.7	2.8	452	0.083
WN_X [8]	32.0	3.1	389	0.070
CrN [9]	19.7	3.4	335	0.058
TiN/AlN* [10]	37.0		400	0.092
TiN/$Cr_{0.37}Al_{0.63}N$ * [11]	28.6	2.5	382	0.074
$Cr_{0.37}Al_{0.63}N$ [11]	25.0	1.3	360	0.069
TiN (Si-Sub) [11]	21.0	2.0	370	0.056
$MoN_{0.5}$/TaN [12]	31.0	3.0	375	0.082
TiN/CrN [13]	24.2	2.0	320	0.075
Ti-Si-N [14]	34.0	3.0		
CrN/TiN [15]	33	2.5		
MoN* [16]	31.9	2.7	424	0.076
$TiMoN_x$* [16]	31.4	3.2	470	0.065
TiN/$MoN_{0.5}$ *[16]	34.8	4.1	446	0.078
ZrN[17]	13.1	1.7	290	0.045
CrN [18]	19.4	3.0	335	0.057
TiN/SiO [19]	12.1	1.9	162	0.076
CrAlN [20]	36.5	3.2		
CrAlSiN [20]	32.0	2.2		
TiAlN [21]	34.0	2.7	470	0.073
(Al,Ta,Ti,V,Zr)N [22]	30.7	2.4	433	0.070

(Al,Ta,Ti,V,Zr)N -Si [22]	29.2	2.3	326	0.089

Supplementary Table 2: A summary of the semi-empirical fracture toughness by DFT calculation. Semi-empirical elastic properties predict the ductility of the material, which can be estimated by the Cauchy pressure (c_{12}-c_{44}) and the shear-to-bulk modulus ratio G/B.

		**B (GPa)**	**G (GPa)**	**E (GPa)**	**B/G**	**c_P**
**Non-stoichiometric**	$TiN/WN_{0.5}$ [8]	297	71	197	4.1	153
	$Ti_{0.5}W_{0.5}N_{0.5}$ [23]	251	100	267	2.5	83
	$MoN_{0.5}/NbN$ [24]	295	99	268	2.95	154
	$MoN_{0.5}/TaN$ [24]	302	106	285	2.84	151
	$MoN_{0.5}$ [16]	300	124	326	2.4	90
	$Ti_{0.5}Mo_{0.5}N$ [16]	289	117	310	2.45	88
	$Ti_{0.5}Mo_{0.5}N_{0.5}$ [16]	227	88	233	2.57	80
	$TiN/MoN_{0.5}$ [16]	285	124	326	2.29	91
	$HfN/WN_{0.5}$ [24]	289	66	183	4.34	168
	$ZrN/WN_{0.5}$ [24]	269	60	168	4.54	159
	$HfC/WN_{0.5}$ [24]	260	122	318	2.12	95
	$TaC/WN_{0.5}$ [24]	324	113	304	2.85	142
	$ZrC/WN_{0.5}$ [24]	235	101	266	2.32	111
**Stoichiometric**	$Ti_{0.875}W_{0.125}N$ [23]	286	171	427	1.66	-5
	$Ta_{0.75}N/TiN$ [24]	277	173	431	1.58	-9
	AlN/HfN [24]	235	167	406	1.40	-17
	AlN/TiN [24]	263	192	464	1.36	-56
	AlN/VN [24]	281	181	446	1.56	-2
	AlN/ZrN [24]	188	129	315	1.47	19
	HfN/TiN [24]	266	170	420	1.56	-18
	HfN/ZrN [24]	260	154	385	1.69	-11
	TiN/ZrN [24]	252	158	392	1.58	-13
	HfC/AlN [24]	192	145	347	1.31	-7
	HfC/TiN [24]	250	181	438	1.36	-52
	HfC/VN [24]	220	90	238	2.43	73

	HfC/ZrN [24]	244	174	422	1.40	-44
	TaC/AlN [24]	281	202	488	1.388	-50
	HfC/TiC [24]	240	176	425	1.36	-55
	HfC/ZrC [24]	231	169	407	1.36	-51
	TiC/ZrC [24]	238	162	393	1.40	-43
	TaC/ZrC [24]	266	190	461	1.38	-53

Supplementary References

1. Motz C, Schöberl T, Pippan R. Mechanical properties of micro-sized copper bending beams machined by the focused ion beam technique. *Acta Materialia* **53**, 4269-4279 (2005).

2. Konijnenberg PJ, Zaefferer S, Raabe D. Assessment of geometrically necessary dislocation levels derived by 3D EBSD. *Acta Materialia* **99**, 402-414 (2015).

3. Ma X*, et al.* Shear strain gradient in Cu/Nb nanolaminates: Strain accommodation and chemical mixing. *Acta Materialia* **234**, 117986 (2022).

4. Gao H, Huang Y, Nix WD, Hutchinson JW. Mechanism-based strain gradient plasticity— I. Theory. *Journal of the Mechanics and Physics of Solids* **47**, 1239-1263 (1999).

5. Chen Z*, et al.* Atomic-scale understanding of the structural evolution of TiN/AlN superlattice during nanoindentation— Part 1: Deformation. *Acta Materialia* **234**, 118008 (2022).

6. Chen Z*, et al.* Atomic insights on intermixing of nanoscale nitride multilayer triggered by nanoindentation. *Acta Materialia* **214**, 117004 (2021).

7. Zhang Q, Zhang LY, Jin CH, Wang YM, Lin F. CalAtom: A software for quantitatively analysing atomic columns in a transmission electron microscope image. *Ultramicroscopy* **202**, 114-120 (2019).

8. Buchinger J*, et al.* Toughness enhancement in TiN/WN superlattice thin films. *Acta Materialia* **172**, 18-29 (2019).

9. Best JP, Zechner J, Wheeler JM, Schoeppner R, Morstein M, Michler J. Small-scale fracture toughness of ceramic thin films: the effects of specimen geometry, ion beam notching and high temperature on chromium nitride toughness evaluation. *Philosophical Magazine* **96**, 3552-3569 (2016).

10. Fallmann M, Chen Z, Zhang ZL, Mayrhofer PH, Bartosik M. Mechanical properties and epitaxial growth of TiN/AlN superlattices. *Surface and Coatings Technology* **375**, 1-7 (2019).

11. Buchinger J*, et al.* Fracture toughness trends of modulus-matched TiN/(Cr,Al)N thin film superlattices. *Acta Materialia* **202**, 376-386 (2021).

12. Hahn R*, et al.* Mechanistic study of superlattice-enabled high toughness and hardness in MoN/TaN coatings. *Communications Materials* **1**, 62 (2020).

13. Hahn R, Bartosik M, Soler R, Kirchlechner C, Dehm G, Mayrhofer PH. Superlattice effect for enhanced fracture toughness of hard coatings. *Scripta Materialia* **124**, 67-70 (2016).

14. Bartosik M*, et al.* Fracture toughness of Ti-Si-N thin films. *International Journal of Refractory Metals and Hard Materials* **72**, 78-82 (2018).

15. Löfler L, Hahn R, Mayrhofer PH, Bartosik M, Holec D. Mechanical properties of CrN-based superlattices: Impact of magnetism. *Acta Materialia* **218**, 117095 (2021).

16. Gao Z, Buchinger J, Koutná N, Wojcik T, Hahn R, Mayrhofer PH. Ab initio supported development of TiN/MoN superlattice thin films with improved hardness and toughness. *Acta Materialia* **231**, 117871 (2022).

17. Zhao XJ, Chen DL, Ru HQ, Zhang N. Zirconium nitride nano-particulate reinforced Alon composites: Fabrication, mechanical properties and toughening mechanisms. *Journal of the European Ceramic Society* **31**, 883-892 (2011).

18. Best JP*, et al.* A comparison of three different notching ions for small-scale fracture toughness measurement. *Scripta Materialia* **112**, 71-74 (2016).

19. Daniel R*, et al.* Fracture toughness enhancement of brittle nanostructured materials by spatial heterogeneity: A micromechanical proof for CrN/Cr and TiN/SiOx multilayers. *Materials & Design* **104**, 227-234 (2016).

20. Drnovšek A, Vo HT, de Figueiredo MR, Kolozsvári S, Hosemann P, Franz R. High temperature fracture toughness of single-layer CrAlN and CrAlSiN hard coatings. *Surface and Coatings Technology* **409**, 126909 (2021).

21. Bartosik M, Rumeau C, Hahn R, Zhang ZL, Mayrhofer PH. Fracture toughness and structural evolution in the TiAlN system upon annealing. *Sci Rep* **7**, 16476 (2017).

22. Hahn R, Kirnbauer A, Bartosik M, Kolozsvári S, Mayrhofer PH. Toughness of Si alloyed high-entropy nitride coatings. *Materials Letters* **251**, 238-240 (2019).

23. Buchinger J, Koutná N, Kirnbauer A, Holec D, Mayrhofer PH. Heavy-element-alloying for toughness enhancement of hard nitrides on the example Ti-W-N. *Acta Materialia* **231**, 117897 (2022).

24. Koutná N, Brenner A, Holec D, Mayrhofer PH. High-throughput first-principles search for ceramic superlattices with improved ductility and fracture resistance. *Acta Materialia* **206**, 116615 (2021).
