# Supplementary material for: Large mechanical properties enhancement in ceramics through vacancy-mediated unit cell disturbance
Source: Nat Commun. 2023 Dec 16;14:8387. doi: 10.1038/s41467-023-44060-x (PMC10725508; doi:10.1038/s41467-023-44060-x)
Supplement: Supplementary file 3 — Description of Additional Supplementary Files [file 41467_2023_44060_MOESM3_ESM.docx]

**Description of Additional Supplementary Files**

File Name: Supplementary Movie 1

Description: Ab initio molecular dynamics (AIMD) simulation of tensile deformation of TiN and WN_0.5_/TIN at 300 K. The tensile direction is along the <110 > direction.

File Name: Supplementary Movie 2

Description: Ab initio molecular dynamics (AIMD) simulation of tensile deformation of TiN and WN_0.5_/TIN at 300 K. The tensile direction is along the <100 > direction.
